# Attracting the next generation of radiologists: a statement by the European Society of Radiology (ESR)

**DOI:** 10.1186/s13244-022-01221-8

**Published:** 2022-05-04

**Authors:** European Society of Radiology (ESR), Jim Zhong, Rosemary Ho, Sofia Gourtsoyianni, Laura Oleaga, Carlo Catalano, Minerva Becker, Vicky Goh

**Affiliations:** European Society of Radiology (ESR), Vienna, Austria

**Keywords:** Education (Medical, Undergraduate), Radiology (Interventional), Diagnostic imaging, Preceptorship

## Abstract

With demand increasing each year for diagnostic imaging and imaging guided interventions, it is important for the radiology workforce to expand in line with need. National and international societies such as the European Society of Radiology have an important role to play in showcasing the diversity of radiology, and highlighting the key role radiologists have in patient care and clinical decision-making to attract the next generation of radiologists. Medical students are an important group to engage with early. Meaningful exposure of undergraduates to radiology with an integrated programme and clinical placements in radiology is essential. Elective courses and dedicated 1-year Bachelor or Masters imaging programmes provide medical students with an opportunity for more in-depth study of radiology practice. Undergraduate radiology societies improve opportunities for engagement and mentorship. Innovations in imaging such as augmented-reality simulation and artificial intelligence and image-guided intervention also offer exciting training opportunities. Through these opportunities, students can gain insight into the wide variety of career opportunities in radiology.

## Key points

- Faculties of medicine, but also national and international societies such as the European Society of Radiology have an important role to play in showcasing radiology and attracting the next generation of radiologists.
- Radiologists should participate in the design of local undergraduate programmes and dedicated Bachelor or Masters imaging programmes can provide an opportunity for in depth study of radiology practice, latest innovations and research.
- Mentorship of medical students by radiologists can improve engagement in the specialty, as well as stressing the importance of the radiologist’s role for diagnosis and clinical decision making and discussing other job aspects that require clinical skills, e.g., interventional procedures or involvement in multidisciplinary tumour boards.
- Undergraduate radiology societies enable greater engagement with medical students to promote the specialty.

## Introduction

Diagnostic and interventional radiologists are central to healthcare systems by providing comprehensive diagnostic services and minimally invasive treatment options for all organs systems, and namely for cancer, cardiovascular disease and stroke, the top causes of mortality and morbidity [1]. Despite the expanding role of clinical imaging through technological innovation, undergraduate radiology education in medical schools is often integrated in the modules of other clinical disciplines, and sometimes delivered by teachers from other specialties. A rethink is required to ensure newly qualified doctors are well-informed of the important role that radiology plays in modern day medicine and of the opportunities and career choices offered by this specialty.

In some countries, the radiology workforce is also increasingly unable to meet the increasing demands for imaging. For example, in the United Kingdom, the 2021 Royal College of Radiologists workforce census has again highlighted a projected shortfall of 44% by 2025, equating to an extra 3,613 radiologists to meet the demand [2]. Similar radiologist shortages are expected across other European countries, e.g., France due to the combination of an aging population and retiring senior consultants [3]. The time is now to reach out to the next generation of radiologists and provide much needed inspiration. Radiology provides a variety of different career options, from hands-on interventional radiology to imaging of molecular processes with functional and nuclear imaging, all equally rewarding in their own right.

Undergraduate representation of radiology remains highly variable and this is likely to be a major factor in whether students consider radiology as a potential career option. A recent survey of over 2700 medical students across Europe found two thirds of respondents had not received any formal exposure to interventional radiology during their undergraduate programme although 95% of students felt that there was a bright future for radiology and almost one in five were likely or very likely to pursue a career in interventional radiology [4].

Despite the variation in exposure to radiology at the undergraduate level, radiology remains one of the most desirable specialties for junior doctors to apply to in many countries, with one of the highest number of applications per vacancy [5]. While medical school undergraduate curriculums have engaged radiology in delivering anatomical knowledge and focussed on the use of imaging in a problem-based approach for doctors to practice safely and effectively; radiologists need to better engage with medical students to ensure these doctors of the future consider radiology as a career. Further initiatives are now required. In this white paper, examples of integrated radiology training schemes, selected to highlight differences in good practice from institutions in five different European countries and to spotlight their strategies for engagement, along with the role of national radiology bodies and undergraduate radiology societies in attracting the next generation of radiologists will be discussed. The importance of mentorship and future focus areas for medical students will also be highlighted.

## Integrated undergraduate radiology training

The European Society of Radiology (ESR) has recently published a new edition of the ESR Curriculum for Undergraduate Radiological Education in order to provide a full and comprehensive update, aimed at aligning the Curriculum with the current state of undergraduate education in Europe. Its contents are widely endorsed by the competent European associations [6]. In many medical schools throughout Europe, radiological anatomy is typically introduced from Year 1, through anatomy teaching sessions, which allow the students to relay what they have learnt in the dissection laboratory to cross-sectional computed tomography (CT) or magnetic resonance imaging (MRI). Ultrasound (US) may also be used to teach abdominal anatomy, which is a practical and effective way for students to learn, and gain confidence in image interpretation skills.

Nevertheless, a review of undergraduate radiology teaching found that one area in need of further development was dedicated radiology clinical placements, which may not be available widely [7]. Junior doctors have also expressed that more undergraduate radiology teaching would be beneficial, and ideally this should be delivered by radiologists in either small group sessions or integrated into clinical teaching [8]. An integrated radiology education, that occurs throughout the medical curriculum, and also includes a stand-alone clinical radiology placement, may increase exposure of medical students to radiology.

For example, at the University of Barcelona during the third year of training, medical students have a compulsory rotation in the radiology department. The rotation is designed as an overview of the different areas of radiology conducted by radiologists assigned to each of the organ-based radiology subspecialties. There are three types of educational sessions:

- Preparatory sessions to learn to recognise the anatomy and interpret the semiology of the chest, central nervous system, abdomen, pelvis and musculoskeletal system;
- Practical sessions in which students observe the acquisition of different imaging exams including CT, MRI, mammography, X-Rays, fluoroscopy, angiography and ultrasonography (US) to learn how these studies are performed, to understand the workflow and the clinical-radiological interaction in the diagnostic interpretation process. In addition, medical students can practice to identify normal anatomic structures on a dedicated US machine under the supervision of a radiologist. This is the most popular activity with the best student rating.
- An interactive case-based teaching session presented by a radiologist with a self-assessment test.

In the last three years, online material with recorded sessions that include basic and general concepts have been incorporated into the teaching programme. These are preparatory sessions the students have to go over in advance to become familiar with radiology before starting the specific rotation.

Another example of early student immersion into radiology includes the approach implemented at the University of Leeds, where during the third year of training, all medical students spend a one-week rotation in the radiology department. At the start of the week, students take part in an interactive case-based teaching session presented by a team of radiology residents. This follows the journey of a fictional patient who requires multiple different imaging tests and interventions, to highlight common radiological investigations and explain the rationale behind their use. Relating this back to the patient’s clinical presentation is vital to aid in the student’s understanding. The rest of the week is designed so that the medical students may observe a variety of diagnostic reporting in different modalities, and also intervention lists in ultrasound and fluoroscopy. They have a workbook to complete with set tasks including identification of normal anatomy on X-ray and computed tomography studies. Seeing the day-to-day work is important for students to gain insight into what a radiologist’s job involves. Radiology may be seen as a faceless specialty by some students who never have the opportunity to engage with the specialty however once they do, most realise how rewarding, stimulating and enjoyable it is.

At Sapienza University of Rome, and other institutions in Italy, radiology is generally studied in the second semester of the 4th year of medical school. Together with lectures, there are six weekly hours of practical activities, during which medical students attend the department. These are not predefined but, in most universities, medical students rotate to follow the execution of all radiology exams (X-ray, US, CT and MRI), attend and sometimes perform post-processing activities, attend reporting sessions and case-based interactive teaching sessions.

At the National and Kapodistrian University of Athens in Greece since 2020 medical students are taught Radiology during two semesters, the second semester of the 3rd year and the first semester of the 4^th^ year, which has enabled the curriculum to be enriched and for new lecture topics such as artificial intelligence to be added. In the preCovid-19 era medical students had obligatory small group clinical rotations of four-hour duration during each semester where they were given the tour of the department, were shown the different available imaging modalities (including X-rays, US, CT, MRI, nuclear medicine department and IR suite) and a selection of day cases were also discussed, while in the ultrasound department they were too given the opportunity to scan volunteers under supervision. In addition, formal lectures taking place in the amphitheatre held by academic radiologists, were followed every time by case-based workshops prepared and given by radiology residents (supervised by academic radiologists). During the pandemic the clinical rotation unfortunately no longer took place but all lessons were taught online and interactivity was encouraged via embedded question polls.

At the University of Geneva in Switzerland during the past twenty years medical students have been taught Radiology using a combination of ex cathedra lectures, interactive teaching in small groups and—since the COVID-19 pandemic—also additional online courses. In addition, 6th-year medical students may choose to enrol in monthly or bimonthly rotations in the radiology department during which they are fully immersed and exposed to the different areas of clinical radiology, following the activities of radiology residents, attending multidisciplinary conferences and participating in the daily structured teaching sessions for residents. This enables academic staff to transmit their excitement and enthusiasm about the ever-evolving field of radiology, to highlight the radiologist’s role in patient care and to discuss other job aspects beyond image interpretation, e.g., interventional procedures or involvement in multidisciplinary tumour boards.

## Dedicated bachelors and masters programmes in imaging

Dedicated one-year Bachelor and Masters programmes focussed on imaging are relatively small programmes and are not universally available throughout Europe but do provide medical students or junior doctors with the opportunity for more in depth study of imaging including state-of-the-art technology; opportunities to undertake an imaging research project; offer greater engagement with radiologists delivering these programmes; and experience of the workings of a radiology departments over a longer period than current undergraduate programmes.

Examples of such programmes are those offered by Sapienza University of Rome in Italy; University of Utrecht in the Netherlands; National and Kapodistrian University of Athens in Greece; and University of Manchester, University of Leeds, University College London and King’s College London in the United Kingdom. Initial data from one programme suggest that medical students who have spent a significant amount of time in radiology department over the year have considered radiology as a future career. Although such dedicated master programmes focussed on imaging may not be widely available, the possibility to choose among a variety of elective tutorials related to radiologic topics or to accomplish a master thesis in the field of radiology as part of the regular curriculum may be a good alternative to attract young future doctors.

At the University of Geneva, as well as in other Swiss Universities, students in the 2nd or 3rd year of the master programme may choose to accomplish their master thesis with a senior member of the academic radiology staff. In addition, elective tutorials on radiology topics are already offered in some Swiss universities early in medical school, typically during the first and second years of the undergraduate medical studies (2nd and 3rd bachelor years). Through this early exposure, many myths, e.g., “dark room”, “lots of physics” and “hardly any patient contact” can be dispelled.

## Undergraduate radiology societies

Whilst it is important for radiologists to promote the specialty to undergraduates, it is also meaningful for students to see their peers engaged in learning more about the specialty. Undergraduate radiology societies comprise a committee of interested and motivated undergraduate students to promote Radiology to their peers. There has been an expansion of undergraduate radiology societies in the United Kingdom in the last five years, with support also provided by the Royal of College of Radiologists. The undergraduate radiology societies organise and host events, liaising with the local Radiology department to do so. A stepwise framework of how to start a society with suggestions for types of events to organise are given in this section.

### Founding an undergraduate radiology society: the process

Medical schools differ in their stipulations and paperwork. Founders should be familiar with local procedures and deadlines for submitting relevant paperwork. The following comprise generalised considerations founders should consider:

- Society ratification—which student bodies to ratify under, necessary paperwork, duration of the ratification procedure, appeals process to an unfavourable outcome
- Committee structure
- Finances—sources of income to fund events. Examples of funding streams include membership joining fees or sponsorship from external organisations.

Different medical schools will have different requirements on the minimum number of students required to form an undergraduate radiology society and the types of roles expected. Core positions typically include:

- President—to oversee the running of the society
- Treasurer—to manage the society's finances, obtain funding, complete any relevant documentation for reimbursement and raise capital
- Secretary—to appropriately schedule and book rooms for meetings and events; write meeting minutes.

The committee may be expanded depending on interest and need. Several examples are as follows:

- Vice President—to deputise the President
- Education Leads—to identify learning objectives and relevant undergraduate curricula, organise timelines, tutors and a programme
- Phase Leads—to represent the interests of the relevant student cohort
- Event Coordinator—to plan and organise events
- Social Media Lead—to advertise on and manage the society's social media accounts
- Conference Lead—to assemble and lead a taskforce; organise the conference programme
- Honorary President—to advise the committee and provide expertise; this is usually a Radiology registrar or consultant.

The committee should have input from all year groups to optimise student engagement and address unmet needs. It would be desirable if committee members represented all year groups, but some undergraduate radiology societies may choose to allow external advisory roles or run student surveys to match events to demand.

### Types of events to organise

It is desirable for the undergraduate radiology societies to meet before the start of the academic year to plan events for the year and mark out holidays and examination periods. This is particularly pertinent for conferences and ‘series’ events consisting of multiple events under a particular theme. In the case of the latter, advanced planning ensures a logical sequence of events. Spreading out events through the year is advised to account for unexpected changes or the addition of events on an ad hoc basis. A database of contacts should also be assembled at the earliest instance as finding available speakers for events may be challenging on occasion. Contact details may be available on local hospital or university websites.

#### Revision tutorials

Revision tutorials are popular but should not be a replacement for formal teaching. Ideally, the undergraduate radiology societies should work with the local radiology departments and medical schools to arrange revision sessions and tutors. The content for an effective tutorial should be tailored to the appropriate level and account for all stages of training, both at the pre-clinical and clinical levels. Students at the pre-clinical level may benefit more from applied radiological anatomy. Students at the clinical level may benefit more from the following:

- General pathology
- Approaches to interpreting images
- How diagnostic and therapeutic radiology fits into common or acute treatment algorithms
- Appropriateness and considerations when requesting scans.

Like formal teaching organised by the medical school, revision sessions should also encompass a broad range of specialties and imaging modalities and be adapted to any demand for specific sessions.

#### Careers

Visibility to radiology is generally poor with many undergraduates who are unaware of the patient/diagnostic imaging workflow and procedures performed by radiologists. Events showcasing the different subspecialty roles (e.g. “A Week in the Life of…”) may help students make more informed decisions regarding their careers. It is certain that junior doctors will encounter patients during their general medical or surgical training, who have received imaging or had IR procedures performed therefore knowledge of the indication for these is beneficial. Events elucidating the training pathway, application process and interviews are also of equal value, particularly students who want to build their curriculum vitae early given that radiology is becoming increasingly competitive.

#### Symposia and conferences

Undergraduate radiology societies may also choose to organise an undergraduate symposium or conference, typically at the national level. Although running student conferences can be labour-intensive and expensive, they may improve the society’s visibility, provide good networking opportunities, and improve recruitment of motivated candidates into the specialty. These events usually require forward planning so early assembly of a task force is crucial. Considerations include:

- Logistics—location, duration, date and time
- Programme—talks, workshops, speakers
- Finances—sponsorships, reimbursement for travel, ticket prices.

Some undergraduate radiology societies may choose to encourage abstract submissions for posters and presentations, which if advertised early enough, may encourage the output of more Radiology-related projects. Equally, essay competitions where prizes may be awarded may increase student engagement and encourage students to be more aware of current issues facing the specialty.

#### Finances and accounts

Prior to the start of the academic year, the treasurer and president should be aware of where the undergraduate radiology society’s accounts are stored, who is involved in the process of accessing funds and how it is done. For established undergraduate radiology societies, the incumbent treasurer should also provide an adequate handover of this process and the amount of remaining funds in the account.

#### Streams of revenue and sponsorships

The undergraduate radiology societies must be familiar with the potential sources of funding and is highly dependent on the individual medical school. This may include bodies to directly sponsor individual events, or a lump sum may be given to the undergraduate radiology societies by the medical school or equivalent governing body. In the case of the latter, the undergraduate radiology societies, typically newly founded undergraduate radiology societies, may be asked to provide evidence of interest to receive funding. Instrumenting society or event health metrics provides unequivocal evidence of engagement.

Undergraduate radiology societies, particularly newly founded and early-stage societies, may also wish to have a steady source of revenue. Examples include charging ticket fees for individual events or following membership models, whereby the undergraduate radiology society charges an upfront payment in exchange for discounted or free services for the year. Pricing, timing, and effect on student engagement should be accounted for. Trade-offs should be rationalised at the earliest instance and in the context of local, pre-existing financial structures to ensure the undergraduate radiology societies’ long-term sustainability.

#### Reimbursement

The undergraduate radiology societies should also familiarise themselves with the process of reimbursement. It may be the case that a committee member pays “out of pocket” initially, so digital or physical copies of receipts should always be documented.

## Undergraduate radiology teaching

Undergraduate radiology teaching should be led mainly by radiologists. This may be obvious, however, imaging crosses over to many other specialties in medicine and surgery. Whilst these professionals may be competent at interpreting basic radiological images, it is important that radiologists design and lead these teaching programmes and provide a level of quality assurance for the material. Radiologists’ rigorous training in anatomy, physiology, pathology and imaging physics makes them more suited to provide a well-rounded educational experience. Local departments should also strive for protected teaching time for trainees and consultants in order for them to contribute meaningfully and consistently. Medical schools should also ensure adequate feedback and certificates of teaching are provided to help the teachers demonstrate this commitment at their annual appraisals and show how the teaching is being dynamically improved.

### Structure and implementation

**Fig. 1:**
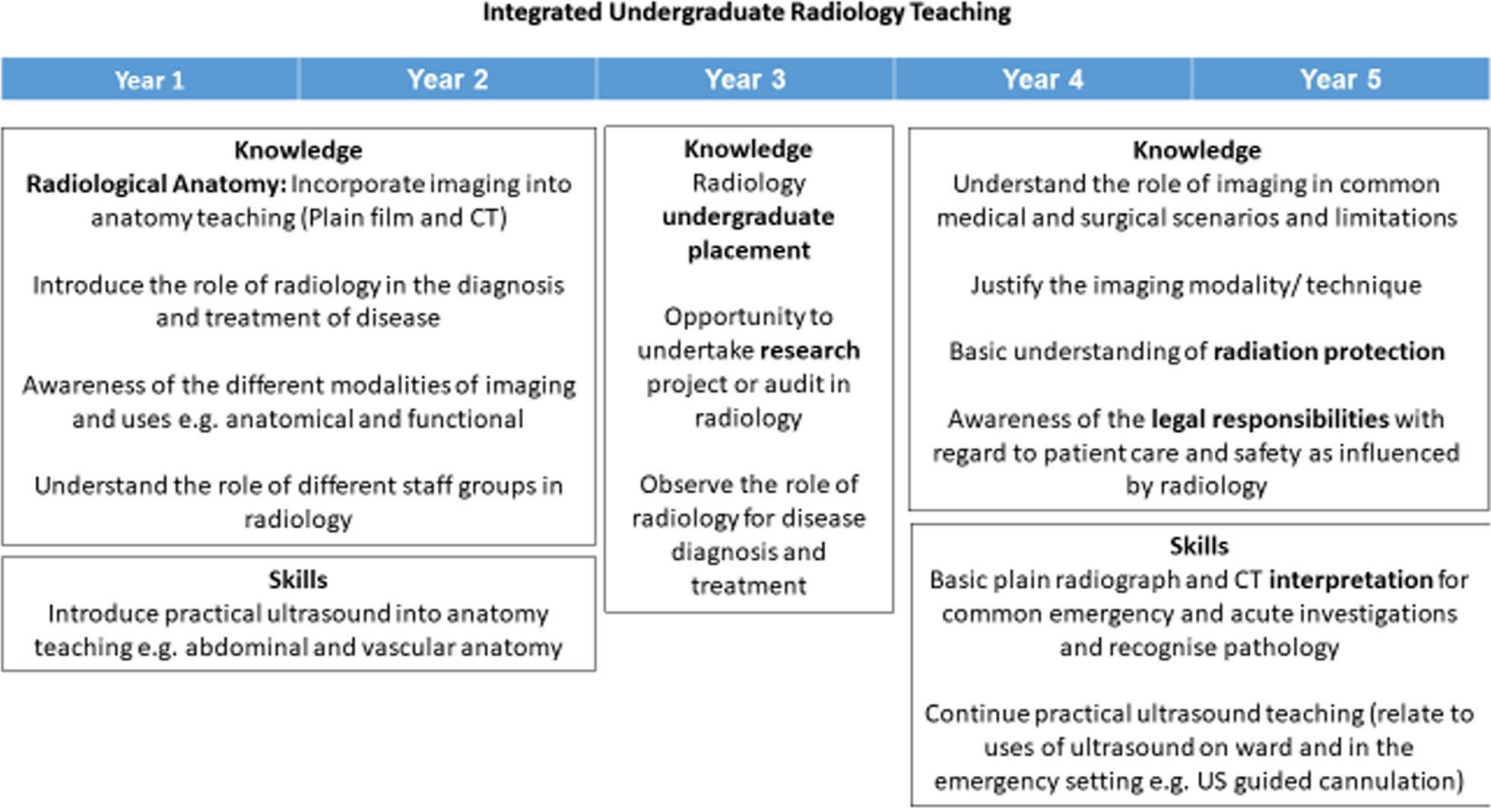
Integrated undergraduate radiology teaching model

Radiology teaching should be integrated and present in all stages of undergraduate training. Students in their pre-clinical phases of training would benefit most from applied radiological anatomy. A potential integrated undergraduate radiology teaching model shown in Fig. 1 is tailored towards the required knowledge at each stage of medical school.

### Teaching environment

The COVID-19 pandemic has accelerated the shift to virtual online teaching, allowing teaching sessions to be more accessible, wider reaching and convenient for students and tutors. Promotion of educational activity organised through undergraduate societies on social media platforms such as Twitter and Facebook have also inspired more groups to collaborate, allowing for networking opportunities and professional development.

## Mentorship

Continuous and strong mentorship led by radiologists-in-training or more senior radiologists should be present in the early stages of undergraduate training, allowing them to experience the breadth of career options and make more informed decisions [9]. Mentors must work to change the stereotype of radiologists, and showcase their typical workflow, amount of patient contact and the clinical importance of the field by explaining how both diagnostic and therapeutic radiology fits into management algorithms. The specialty should be highlighted as an engaging and dynamic field, with high job satisfaction, reasonable work-life balance and intellectual stimulation.

Women and ethnic minorities are still underrepresented in Radiology. It has been suggested that women radiologists holding visible leadership roles and serving as role models may play an important role in recruiting women in radiology residency programmes [10]. Where possible, having a mentor with a similar gender and/or ethnic background to the mentee may help dispel any myths and provide advice on common issues facing a particular demographic (such as raising families in the case of women in radiology) [11, 12].

Mentorship requires a consistent presence and may be implemented through a variety of streams including:

- Teaching—this is an opportunity to inspire students with the content of teaching or through innovative teaching methods. Mentorship may also involve allowing the student to be involved with teaching and offering feedback or through designing new teaching sessions.
- Full time clinical placements should be lobbied for and offer undergraduates the opportunity to attend multidisciplinary team meetings, tumour boards, interventional procedures if available and small groups reporting
- Opt-in student mentorship programs—as an adjunct to full-time clinical placements and may be co-organised in part with undergraduate radiology societies to increase student engagement and reach

Of note, radiologists should be conscious of how students’ personal interactions influence interest in their careers. Radiologists should maintain frank discussions about the merits and problems facing the specialty, but should be enthusiastic, helpful and receptive to the student.

## Future focus areas for medical students

### Professional image of radiology as a clinical discipline

There is a common misconception of the professional image of the radiologist as someone with advanced technical skills who evaluates images on a computer screen but who has little or no patient contact and with very limited impact on clinical decision making. This may not only lead many valuable candidates to avoid radiology as specialty, but also attract candidates into the specialty whose interest is purely technical as well as those who are uncomfortable with responsibilities in the clinical decision process. It appears therefore important to promote the clinical relevance of the radiologist, and to expose undergraduate students not only to those areas of radiology where contact with patients in naturally inherent, eg, clinical mammography, ultrasonography, interventional radiology or advanced magnetic resonance imaging, but also to let them assist in multidisciplinary decisional meetings where radiologists participate directly in clinically relevant processes.

### Data science: artificial intelligence and big data

Another misconception, often enhanced by certain public media, is that artificial intelligence will replace radiologists in a foreseeable future. Most medical students may be generally aware that artificial intelligence is becoming increasingly commonplace in healthcare, and that it is also increasingly used in many other clinical specialties other, e.g, ophthalmology, cardiology or dermatology. Nonetheless, it is important to explain to undergraduate students the way in which tools based on machine-learning may be used and integrated in the radiologist’s workflow, enhancing rather than replacing the radiologist’s role.

There is also an increasing emphasis on big data in radiology research and technically enthusiastic undergraduates should be actively encouraged to engage in academic projects related to data management where possible. For example, medical students could assist in projects of data mining or help to compile test and training datasets to be incorporated into machine learning algorithms. This would help their understanding of the field and give them insight into an expanding academic area within radiology. The prospect of presenting research at a conference, publishing a thesis and expanding their professional network may also help motivate them to continue radiology research.

Altogether, radiologists should therefore spearhead efforts to educate undergraduate medical students on emerging technologies relevant to the specialty, and help to provide insight into the opportunities and challenges required to develop a realistic universal framework for artificial intelligence [12, 13]. Of course, inclusion of dedicated curricula on artificial intelligence in the overall undergraduate medical curriculum would help to streamline this process [14].

### Interventional radiology and simulation

Increased access to learning opportunities in interventional radiology during medical school is vital to ensure students are up to date with the variety of therapeutic options interventional radiology increasingly has to offer in acute and chronic disease conditions. As with surgery, early interventional radiology training would benefit from simulation by allowing for skills to be practiced and evaluated in a safe and controlled environment. This would also help to identify the strengths and weaknesses of students and help them make more informed career choices. Previous work suggested that incorporating interventional radiology simulation into undergraduate education could greatly enhance the skill-set of students pursuing interventional radiology and allied interventional specialties such as cardiology, anaesthetics and intensive care, showing the wide applicability of interventional radiology skills and principles for undergraduates [15].

Ultrasound simulation is already an accepted method of training and early adoption could allow students to master basic skills, improve their confidence and enable them to get more educational value from clinical ultrasound experience whilst reducing the impact of service provision on training [16]. With the advent of virtual and augmented-reality simulation, this will allow users to learn in a new way by interacting and manipulating their environment. This immersing virtual world is associated with higher levels of active participation due to increased social, environmental, and personal presence within the learning activity itself [17]. Early barriers to overcome are the high costs and artificial conditions, which could be perceived as unrealistic, therefore predictive performance metrics of using this technology are required to show transferability before wider adoption.

## Conclusions

To inspire the next generation of radiologists, greater promotion of the specialty in medical schools is required. These should include and emphasise aspects related to the clinical relevance of the radiologist in the multidisciplinary clinical process. This can be achieved through integrated radiology teaching and clinical placements and a curriculum that captures the central role that imaging plays in clinical care. The inception of undergraduate radiology societies has boosted interest amongst students and early mentorship is vital to guide students into radiology. Finally, highlighting radiological innovations on the horizon and preparing students for a data driven future in medicine, will hopefully allow students to realise that radiology is one of the most exciting areas to be part of with a bright future.

## Data Availability

Not applicable.

## Abbreviations

CT: Computed tomography
ESR: European Society of Radiology
MRI: Magnetic resonance imaging
US: Ultrasound
